# Relationship between esophageal motility and severity of gastroesophageal reflux disease according to the Los Angeles classification

**DOI:** 10.1097/MD.0000000000015543

**Published:** 2019-05-13

**Authors:** Lan Liu, Shuai Li, Kongxi Zhu, Weihua Yu, Hongjuan Wang, Jianqiang Guo, Hongwei Gao

**Affiliations:** a Department of Gastroenterology, the Second Hospital of Shandong University; b Department of Trauma and Orthopaedics, the Second Hospital of Shandong University, Shandong Province, People's Republic of China.

**Keywords:** esophageal acid exposure, esophageal motility, gastroesophageal reflux disease, Los Angeles classification, lower esophageal sphincter

## Abstract

The current study aimed to investigate the relationship between the severity of gastroesophageal reflux disease (GERD) according to the Los Angeles (LA) classification and esophageal motility using high-resolution manometry (HRM) and 24-hour esophageal pH monitoring.

We examined 124 patients with GERD from January 2016 to June 2018. The LA classification of each patient was determined by endoscopy. HRM was performed by the intraluminal water infusion method. HRM and 24-hour esophageal pH monitoring parameters of the patients were studied and statistically compared.

On HRM examination, GERD symptoms were found to be associated with worsened distal contractile integral (DCI), ineffective esophageal motility (IEM), peristalsis break (PB), lower esophageal sphincter (LES) pressure, and the 4-second integrated relaxation pressure (IRP4s) of LES pressure along with the grade of LA classification, especially in patients having grade C and D GERD who had transverse mucosal breaks. The 24-hour pH monitoring study revealed that patients classified as having grade C or D GERD had an esophageal pH < 4.0 for a longer time than those with grade O, A, or B GERD. Similar results were found regarding the duration of the longest reflux event, the number of reflux episodes longer than 5 minutes, and the number of reflux episodes. Patients with higher grade esophagitis had higher De Meester scores, which suggested greater esophageal acid exposure. Hiatal hernia (HH) was more closely related to LES pressure, IRP4s, and acid exposure, whereas DCI, IEM, and PB were not statistically different between patients with GERD with and without HH.

Patients with severe esophagitis may have motor dysfunction not only in the LES but also in the esophageal body, with resulting increased esophageal acid exposure, which causes esophagitis. Low LES pressure might be the main reason that patients with HH develop esophagitis. GERD without HH may be due to a variety of motor dysfunctions.

## 1 Introduction

Gastroesophageal reflux disease (GERD) is one of the most prevalent gastrointestinal diseases and is defined as a condition that develops when reflux of the stomach contents causes troublesome symptoms and/or complications.^[1,2]^ The diagnosis of GERD should be made by integrating the results of multiple examinations, including endoscopy, manometry, and pH monitoring.^[3]^ Hiatal hernia (HH) is a frequent finding in patients with GERD. A previous study has shown that HH significantly increases the incidence of GERD.^[4]^

The Los Angeles (LA) classification of GERD was presented during the World Congress of Gastroenterology in Los Angeles in 1994.^[5]^ In this classification, the term “mucosal break” was introduced to describe mucosal lesions of the esophagus. It replaces traditional terms such as erythema, erosion, edema, and ulcer. A mucosal break was defined as an area of sloughing or erythema clearly demarcated from the adjacent normal-appearing mucosa.^[5]^ According to the LA classification, GERD is divided into 4 grades designated A through D.^[6]^

High-resolution manometry (HRM), capable of monitoring pressure from the pharynx to the stomach together with pressure topography plotting, has been used for clinical diagnosis of functional esophageal disorders and clinical research.^[7]^ HRM has become increasingly important and is now the new worldwide standard for the clinical evaluation of esophageal motility disorders. Twenty four hour esophageal pH (24-hour pH) monitoring, an advanced technique used to study esophageal acid exposure for the previous 24-hour period, was used for the current study of patients with GERD.

To the best of our knowledge, the association of pathophysiological changes in patients with GERD, such as acid exposure, esophagus body motility disorder, and low LES pressure, with the severity of mucosal break according to LA classification, during endoscopy has not been established. We hypothesized that there is a relationship between the severity of GERD and the degree of esophageal disruption of motor function. To prove this hypothesis, we designed this study to analyze the severity of GERD in relation to the degree of esophageal motor function. In this study, HRM was combined with 24-hour pH monitoring analysis and a set of data was obtained to examine the relationship between the LA classification and esophageal motility vs acid exposure to investigate the possible mechanisms of mucosal injury in patients with GERD.

## 2 Methods

### 2.1 Patients

According to the “Guidelines for the Diagnosis and Management of Gastroesophageal Reflux Disease (2013),”^[8]^ the diagnosis of GERD is made using a combination of symptom presentation, objective testing with endoscopy, ambulatory reflux monitoring, and response to antisecretory therapy. Heartburn and regurgitation are the most reliable symptoms for making a presumptive diagnosis based on history alone. We retrospectively analyzed the data of patients with GERD who underwent endoscopic examination, ambulatory 24-hour pH monitoring, and esophageal HRM from January 2016 to June 2018 in the Second Hospital of Shandong University, China. The inclusion criteria were a previous diagnosis of typical reflux symptoms such as heartburn and reflux; response to a proton pump inhibitor; evidence of reflux esophagitis on endoscopic examination; and age 18 to 80 years. The exclusion criteria were previous gastrointestinal surgery, pregnancy, or current medications known to affect gastrointestinal motor function or acid secretion. If patients were taking acid suppression medical therapy daily, we performed all examinations after they had discontinued this medication for at least 72 hour; otherwise, they were excluded from the study. A total of 124 patients with GERD were enrolled in our retrospective study. This study was approved by our Institutional Review Board.

### 2.2 Endoscopic examination

Gastroscopy (GIF H260/H290; Olympus, Tokyo, Japan) was carried out to identify the mucosa status of the gastroesophageal junction and to exclude other organic diseases. Two expert endoscopists performed and investigated every endoscopic evaluation. We defined endoscopic GERD by the LA classification. According to the LA classification, GERD was divided into 4 grades designated A through D. These grades are defined as follows: Grade A: 1 or more mucosal breaks confined to the mucosal folds, each no longer than 5 mm; Grade B: at least 1 mucosal break more than 5 mm long confined to the mucosal folds and not continuous between the tops of 2 folds; Grade C: at least 1 continuous mucosal break between the tops of 2 or more mucosal folds but not circumferential and Grade D: 1 or more circumferential mucosal breaks. If there was no obvious mucosal injury, it was called non-erosive reflux disease, or Grade O.

### 2.3 HRM protocol

The classification scheme for HRM, termed the Chicago Classification (CC), has evolved from conventional criteria and has improved clinicians’ ability to make manometric diagnoses.^[9,10]^ Subsequent research has improved the diagnostic accuracy and utility of classification, resulting in the Chicago Classification v3.0 (CC v3.0) update.^[11,12]^ HRM was performed in the standard fashion with the patient in the supine position after at least a 6-hour fast, using the MMS HRM system (Medical Measurement Systems, the Netherlands). After transnasal placement of the manometry assembly, it was positioned to record pressure from the hypopharynx to the stomach. Our manometric protocol also included a 5-minute period to assess the basal sphincter pressure and 10 swallows of 5 ml normal saline.

### 2.4 HRM data analysis

Analysis of manometric data describes the resting characteristics of the esophageal sphincters and esophageal motor functions initiated by swallowing. The upper esophageal sphincter (UES) and lower esophageal sphincter (LES) are easily identified as zones of higher pressure. The 4-second integrated relaxation pressure (IRP4s) algorithm averages the lowest of these pressures, the nadir pressure, over 4 continuous or discontinuous seconds. Peristaltic integrity is assessed by measuring gaps in the 20-mm Hg contour along the length of the esophagus, between the UES and LES. According to the Chicago classification, a small break measures 2 to 5 cm and a large break measures >5 cm^[9,13,14]^; in the CC v3.0 update, only large breaks were scored.^[11,12]^ The distal contractile integral (DCI), which integrates the length of the smooth muscle esophagus (cm), contractile pressure (mm Hg), and contraction duration, is used to measure the robustness of the peristaltic contractions in the smooth muscle esophagus.^[15]^ Ineffective esophageal motility (IEM) has been re-defined in the CC v3.0 as a swallowing DCI of <450 mm Hg/s/cm.^[16]^

### 2.5 24-hour Esophageal pH monitoring

During the study, the patients consumed an unrestricted diet and took no medications that could interfere with the results. The 24-hour esophageal pH monitoring was conducted using an antimony pH catheter (Orion-Ohmega, the Netherlands). The sensor was positioned 5 cm above the LES. Continuous pH recording was performed for 24 hours.

The De Meester score^[13]^ was used to calculate the following distal pH variables: percentage of total time when pH was <4, longest reflux event, number of reflux events longer than 5 minutes, and number of reflux episodes in 24 hours. A De Meester score of >14.72 was considered to indicate significant esophageal acid exposure.

### 2.6 Statistical analyses

Data analysis was performed using SPSS version 16 software. We used analysis of variance for univariate analysis of single variables. A *P* value < .05 was considered to indicate significance.

## 3 Results

### 3.1 Patient characteristics

A total of 124 patients with GERD were enrolled in this study, and they were divided into 4 groups according to LA classification: grade A, 29 cases; grade B, 17 cases; grade C, 14 cases; and grade D, 7 cases. The 57 patients with no obvious mucosal injury were classified to have grade O. The age of patients in the different groups had no significant association with the grade of esophagitis. The incidence of HH significantly increased with a higher LA grade in patients with GERD (Table 1).

**Table 1:**
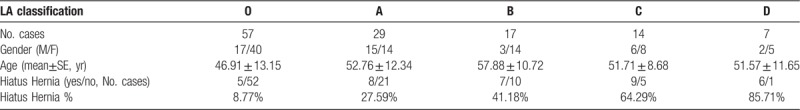
Patient characteristics.

### 3.2 HRM parameters in patients grouped according to LA subgroup

**Figure 1:**
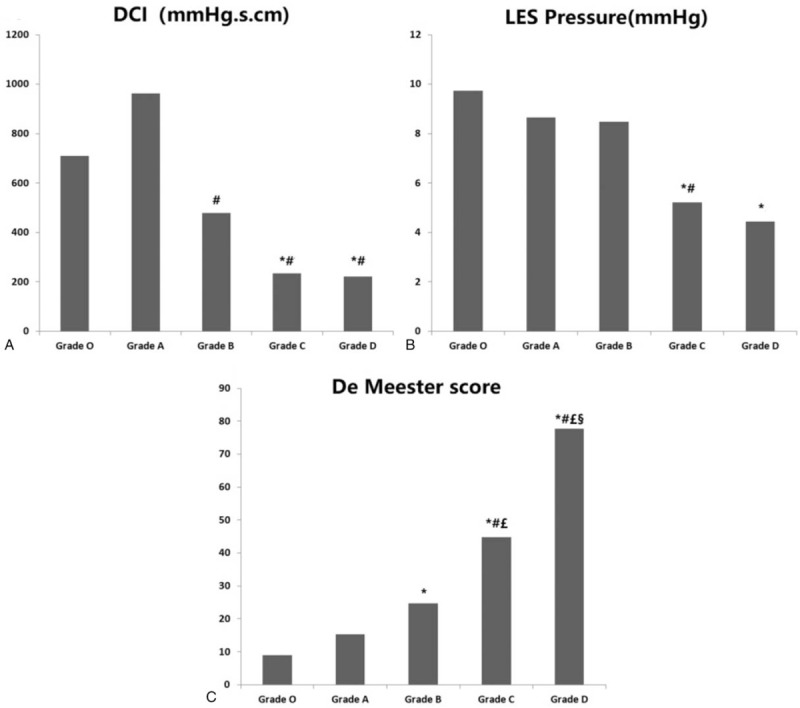
DCI, LES pressure, and De Meester score according to LA subgroup. A: DCI according to LA subgroup. B: LES pressure according to LA subgroup. C: De Meester score according to LA subgroup. ^∗^, ^#^, £, § There was a significant difference when comparing grade O (^∗^), grade A (#), grade B (£), and grade C (§). *P* < .05 indicates a significant difference.

On HRM examination, the patients classified as having grades B, C, and D GERD showed an aggravating tendency in DCI, IEM, PB, LES pressure, and IRP4s of LES pressure, especially in patients classified as having grades C and D, who had high-grade reflux esophagitis (Fig. 1A, B). The HRM results demonstrated an association between a high LA grade and esophageal dysmotility with low LES pressure (Table 2).

**Table 2:**
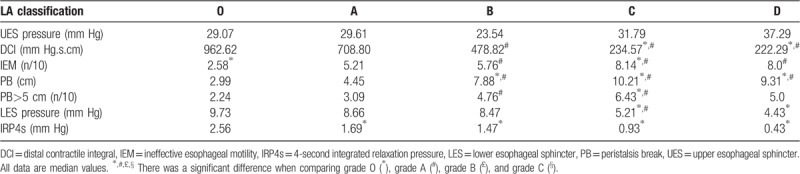
HRM Parameters in patients grouped according to LA subgroup.

### 3.3 Association between esophageal acid exposure and LA subgroup

The 24-hour esophageal pH monitoring study revealed that in patients classified as having grades C or D, the percentage time with an esophageal pH < 4.0 was higher than that in patients classified as having grade O, A, or B. Similarly, the duration of the longest reflux event, the number of reflux episodes, and the number of reflux episodes longer than 5 minutes were higher in patients with grades C or D than in patients with grade O, A, or B esophagitis (Table 3). Patients with severe esophagitis had higher De Meester scores (P < .05), which suggested greater and longer esophageal acid exposure (Fig. 1C).

**Table 3:**
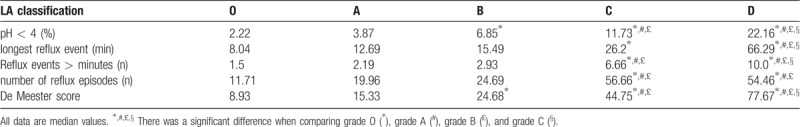
Association between esophageal acid exposure and LA subgroup.

### 3.4 HRM and pH monitoring parameters in patients with and without HH

HRM and 24-hour esophageal pH monitoring parameters in patients with GERD with and without HH were compared (Table 4). HH was more closely associated with LES pressure, IRP4s of LES pressure, the time of longest reflux event, the number of reflux episodes, the number of reflux episodes longer than 5 minutes, and De Meester scores. Patients with GERD and HH had lower LES pressure and experienced greater and longer acid exposure. The differences in DCI, IEM, and PB among patients with GERD with and without HH were not statistically significant.

**Table 4:**
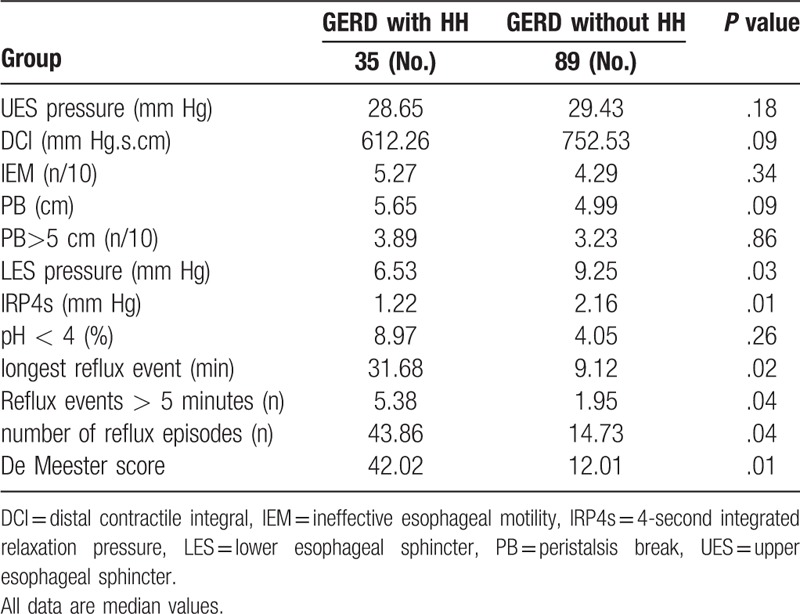
HRM and pH monitoring parameters in patients with and without HH.

## 4 Discussion

GERD has a complex and multifactorial pathogenesis that is associated with esophageal motility, the protective barrier of the esophagus, stomach acidity, and stomach emptying.^[17]^ The primary determinants of GERD severity are a dysfunctional antireflux barrier and impaired esophageal clearance. The antireflux barrier prevents reflux of gastric contents into the esophagus; once the gastroesophageal reflux enters the esophagus, peristalsis occurs to clear the esophagus of the reflux content.^[18]^ The severity of GERD might be expressed by LA classification during endoscopy.

The application of HRM made it possible to measure the pressure pattern throughout the entire length of the esophagus with each swallow, from UES to LES, providing a complete depiction of esophageal motor function.^[19,20]^ HRM has become increasingly important and is now the new worldwide standard for the clinical evaluation of esophageal motility disorders. Esophageal acid exposure can be dynamically observed using 24-hour pH monitoring.

Motility disorders in the esophageal body, as classified by the Chicago classification, focus on the distal esophagus. In HRM, IEM has been found in 21% to 49.4% of patients with GERD.^[21,22]^ Prolonged acid clearance from the esophagus of patients with IEM seems to be the most relevant factor in the development of GERD.^[23]^ The new definition in CC v3.0 has abandoned the concept of a peristalsis defect; however, Ribolsi reported that weak peristalsis with a large break was associated with high acid exposure and delayed reflux clearance in the supine position in patients with GERD.^[24]^ In the current study, on HRM examination, GERD symptoms were found to be associated with worsened DCI, IEM, PB, LES pressure, and IRP4s of LES pressure along with the grade of LA classification, especially in patients classified as having grades C and D GERD who had high-grade reflux esophagitis. The HRM results supported that high-grade patients suffered from esophageal dysmotility and low LES pressure, similar to the findings of previous studies.

Lundell found a significant relationship between the LA classification grade and the 24-hour esophageal acid exposure values.^[6,25]^ In the current study, we examined the characteristics of gastroesophageal acid reflux in patients grouped according to LA classification. Our results found that the percentage of time that the esophageal pH was lower than 4.0 in patients with grades C or D was higher than that in patients with LA grades A or B. Similar results were found for the time of longest reflux event, the number of reflux episodes longer than 5 minutes, and the number of reflux episodes. Patients with higher grade esophagitis had higher De Meester scores (*P* < .05), suggesting greater esophageal acid exposure.

Severe esophagitis (LA grades C and D) with transverse mucosal breaks may cause motor dysfunction not only in the lower esophageal sphincter but also in the esophageal body, which results in longer esophageal exposure to acidic gastric contents, as a recent study demonstrated.^[26]^ Our findings indicated that the decrease in resting LES pressure in patients with grades C and D can reasonably explain the occurrence of the continuous mucosal break between the tops of 2 or more mucosal folds.

Impaired esophageal function has been shown to be the cause of esophageal mucosal damage.^[27]^ The current study confirmed this finding. Low LES pressure was an important etiology of acid reflux, and esophageal motor dysfunction aggravated the acid reflux. Acid reflux and motor dysfunction induced and aggravated esophagitis.

The current study found that HH was closely related to LES pressure, IRP4s, and acid exposure but had no obvious correlation with esophageal body motility. These results verified that low LES pressure might be the main reason that patients with HH experience esophagitis, whereas GERD without HH may be due to a variety of reasons including esophageal body dysmotility, low LES pressure, and acid exposure.

There were also some limitations of this study. First, it was a retrospective study, and we will confirm the results in a prospective study. Second, some patients did not undergo all tests (endoscopy, 24-hour pH monitoring, and HRM), which prevented them from being included in this study and reduced the sample size.

In summary, we retrospectively reviewed the data for esophageal HRM and 24-hour pH monitoring of patients with suspected GERD to observe the possible influence of mechanisms of mucosal injury. We found that patients with severe esophagitis with transverse mucosal breaks may have motor dysfunction not only in the lower esophageal sphincter but also in the esophageal body, with resulting longer and greater esophageal exposure to acidic gastric contents. In patients with GERD and HH, low LES pressure might be the main cause, whereas GERD without HH may be due to a variety of motor dysfunctions.

## Author contributions

**Conceptualization:** Kongxi Zhu.

**Data curation:** Lan Liu, Shuai Li, Kongxi Zhu.

**Formal analysis:** Hongjuan Wang.

**Funding acquisition:** Lan Liu.

**Investigation:** Lan Liu.

**Methodology:** Shuai Li, Kongxi Zhu, Weihua Yu.

**Project administration:** Hongjuan Wang, Jianqiang Guo, Hongwei Gao.

**Software:** Hongwei Gao.

**Validation:** Jianqiang Guo.

**Writing – original draft:** Lan Liu, Shuai Li.

**Writing – review & editing:** Weihua Yu, Hongjuan Wang, Jianqiang Guo, Hongwei Gao.
